# Syrup of Iodide of Iron

**Published:** 1868

**Authors:** 


### SYRUP OF IODIDE OF IRON.

The preservation of this very useful preparation of iron and iodine has engaged the attention of pharmaceutists for several years past.

The American Journal of Pharmacy copies an article from the London Pharmaceutical Journal, written by Thos. B. Groves, F. C. S., giving his experiments with this preparation. To thirty-one fluid ounces of the syrup he adds half fluid ounce dilute phosphoric acid, and says "phosphoric acid is the only acid that can be relied on for the preservation of the syrup of iodide of iron."

